# Linkage of premature and early menopause with psychosocial well-being: a moderated multiple mediation approach

**DOI:** 10.1186/s40359-023-01267-3

**Published:** 2023-08-09

**Authors:** Sampurna Kundu, Sanghmitra Sheel Acharya

**Affiliations:** Centre of Social Medicine and Community Health, School of Social Sciences, Jawaharlal Nehru University, Delhi, 110067 India

**Keywords:** Premature menopause, Early Menopause, Smoking, Depression, Insomnia, Cognition

## Abstract

**Purpose:**

Menopause occurring before the age of 40 is premature and between 40 and 44 years age is early, since the natural age of menopause lies between 45 and 50. The endocrine changes that come with menopause include an erratic decline in estrogen levels which affects the brain. Thus, leading to changes in cognitive function in the longer term due to the menopausal transition. The study aims to explore the effect of premature and early menopause on cognitive health, and psychosocial well-being. The moderated multiple mediation hypothesis of the study is that the effect of premature or early menopause is mediated by depression and insomnia, while all the pathways are moderated by smoking habits.

**Data and Methods:**

The study utilized Longitudinal Aging Study in India (LASI), 2017–2018, Wave 1 data. The sample of 31,435 women were aged 45 and above and did not undergo hysterectomy. A moderated multiple mediation model was used to understand the association between premature or early menopause (X), insomnia (M1), depression (M2), moderator (W), and cognitive health (Y), while controlling for possible confounders.

**Results:**

Premature menopause was negatively associated with cognition (β:-0.33; SE:0.12; p < 0.05), whereas positively associated with insomnia (β:0.18; SE:0.03; p < 0.001) and depression (β:0.25; SE:0.04; p < 0.001). There is a moderating effect of smoking or tobacco consumption has a significant moderating effect on the pathways among premature menopause, depression, insomnia and cognition. When the same model was carried out for early menopause (40–44 years), the results were not significant.

**Conclusions:**

The findings emphasize the fact that smoking is associated with premature menopause, depression and insomnia. Women who experienced premature menopause has lower cognitive scores, depressive symptoms and insomnia symptoms, which were higher among those who consumed tobacco. The study, strongly recommends the dissemination of information on the negative effects of tobacco consumption and making more informed choices to maintain a healthy life. More research into various methods and therapy is needed to determine the relationship between the age of early menopause and their psychosocial well-being.

**Supplementary Information:**

The online version contains supplementary material available at 10.1186/s40359-023-01267-3.

## Introduction

Menopause is an important event that marks the end of a woman’s reproductive period. The majority of women normally attain menopause in the age bracket of 45 and 55 [1]. Some women, due to ovarian insufficiency attains menopause at an early age due to lifestyle factors and hormonal imbalances. Menopause occurring before the age of 40 is premature and between 40 and 44 years age is early, since the natural age of menopause lies between 45 and 50. The cessation of menses is marked by amenorrhea, a rise in gonadotropin levels and oestrogen deficiency [2].

The endocrine changes that come with menopause include an erratic decline in estrogen levels which affects the brain [3]. Thus, leading to changes in cognitive function in the longer term due to the menopausal transition [4].

### Effect of premature failure of ovarian function on cognitive abilities

Evidence of changes in estrogen levels accounting for speculated increased memory complaints during the menopausal transition exists [5, 6]. There are experimental studies that have suggested that estrogen has neurological effects post menopause, the brain atrophy of women accelerates at a faster rate than in men [7, 8]. Given that this fact is still debatable [9, 10], there have been reported positive correlations between endogenous estrogen levels and cognitive function [11, 12]. Also, there have been empirical studies that reported that women who experience premature ovarian failure, be it surgical or natural, have poor verbal fluency and memory in later life. It is associated with an increased risk of psychomotor speed decline [1, 13].

### Mediating role of depression between premature failure of ovarian function and cognition

An emerging public health challenge in the global scenario is depression, which affects people of all ages and is one of the leading causes of morbidity. Stress, adverse life events, biological, psychological, and social factors are all associated with depression [14]. Cognitive impairments interfere with one’s ability to think or focus, including indecisiveness, and memory loss all of which are criterion items for a diagnosis of depression [15]. Postmenopausal hormonal imbalances lead to neurological impacts and evidences show that depression is increased in later life, is menopause is attained prematurely [4, 16].

In addition to the psychological impacts, depression typically affects a person’s life, social relationships, psychomotor abilities, and leads to fatigue and even insomnia [17]. Studies have found that patients suffering from depression had problems distinguishing between similar or identical objects and suffered short-term memory loss [18–20]. Depression is one of the evident symptoms of menopause and attaining menopause prematurely or before the natural age, can lead to amplification of the symptoms [21]. Reinforcing the neurological evidence of premature ovarian failure and cognitive outcomes of depression, we posit that depression could play a mediating role in the associations between premature or early menopause and cognitive impairment.

### Mediating role of insomnia between premature failure of ovarian function and cognition

Ageing comes with difficulties with sleep caused by many factors such as multimorbidity, ageing process, increased medication use and others [22–25]. Insomnia is one of the common symptoms of menopause, along with other features like hot flushes, headache, body weakness, and depression [21].

Evidently, changes in sleep structure with aging are linked to cognitive decline, that is, decreased performance across various cognitive activities, including information processing speed, perceptual speed, executive functioning, concentration and attention, inhibitory functioning, and memory [26]. Per a growing body of literature, sleep facilitates synaptic plasticity, promotes procedural learning processes, and the consolidation of declarative memories and other cognitive functions [23, 27–29]. Thus, treating nocturnal insomnia may enhance cognitive performance and overall quality of life. With the understanding of the possibility of the woman’s early onset of ovarian failure influencing sleep disturbances, we hypothesize that insomnia would mediate the association between premature or early menopause and cognitive impairment.

### Moderating role of smoking among premature failure of ovarian function, insomnia, depression and cognition

Lifestyle habits are a major factor that has an impact on a woman’s reproductive cycle [30]. In recent times, smoking cigarettes and in general tobacco consumption in any form is very common, and also a highlighted factor in damaging the reproductive life [31–33]. Studies have suggested that cigarette smoking leads to menstrual disorders and also has a moderate impact on ex-smokers. These studies showed that those who smoked had more risk of having oligomenorrhea and abnormal menstruation like irregularity and intermenstrual bleeding [34–37]. A 21-year follow-up study by [38], also found that the risk of early menopause is lower for those who do not smoke or have quit smoking.

Large cohort studies have shown that smoking reduces sleep quality and contributes to more insomnia-like symptoms [39–42]. Smoking leads to more severity of menopausal symptoms and poor sleep quality [43]. Along with sleep disturbances, there is also a prevalence of depressive symptoms due to smoking addiction [44–47]. Active smoking has neurotoxic effects on the brain, which leads to an increased risk of cognitive impairment, along with the development of underlying vascular disorders [48]. Longitudinal studies have shown that smoking is a major risk factor for cognitive decline with ageing [49]. Thus, it can be assumed that smoking moderates the pathways among early cessation of menopause, insomnia, depression and cognition.

### Study hypothesis

#### Mediation hypothesis

There is a direct or indirect effect of premature or early menopause on cognitive health through insomnia and depression while controlling possible confounders.

H_a_ : Having premature or early menopause is associated with deteriorating cognitive health.

H_b_ : Having premature or early menopause is associated with higher depression levels, which leads to deteriorating cognitive health.

H_c_ : Having premature or early menopause is associated with higher insomnia symptoms, which leads to deteriorating cognitive health.

#### Moderated-mediation hypothesis

H_d_ : There is a direct or indirect effect of premature or early menopause on cognitive health through insomnia and depression, moderated by smoking (tobacco consumption) while controlling possible confounders.

## Methods

### Data source

The study utilized the Longitudinal Aging Study in India (LASI), 2017–2018, Wave 1 data. It is a multipurpose nationally-representative longitudinal survey that provides rich information on demographics, cognition, mental and physical health, and social networks of the ageing population in India. The survey collected extensive information on individuals’ physical, social and cognitive health (72,250) aged 45 and above across all states and union territories of India (excluding Sikkim). The survey used a multistage stratified area probability cluster sampling design wherein three-stage sampling was conducted for rural areas and a four-stage sampling design was conducted for urban areas to arrive at the eventual units of observation: older adults age 45 and above and their spouses irrespective of age. The goal was to select a representative sample in each stage of sample selection. India is a union comprising 30 states and 6 union territories with a population of 1,211 million [50] at the time of the survey. India’s states vary significantly with respect to geography, culture, population size, health conditions, demographics, and socio-economic characteristics.

The research design of the study is cross-sectional and descriptive, where the study attempts to analyze the secondary data to understand the effect of premature and early menopause on the psychosocial well-being of women in later ages.

**Fig. 1:**
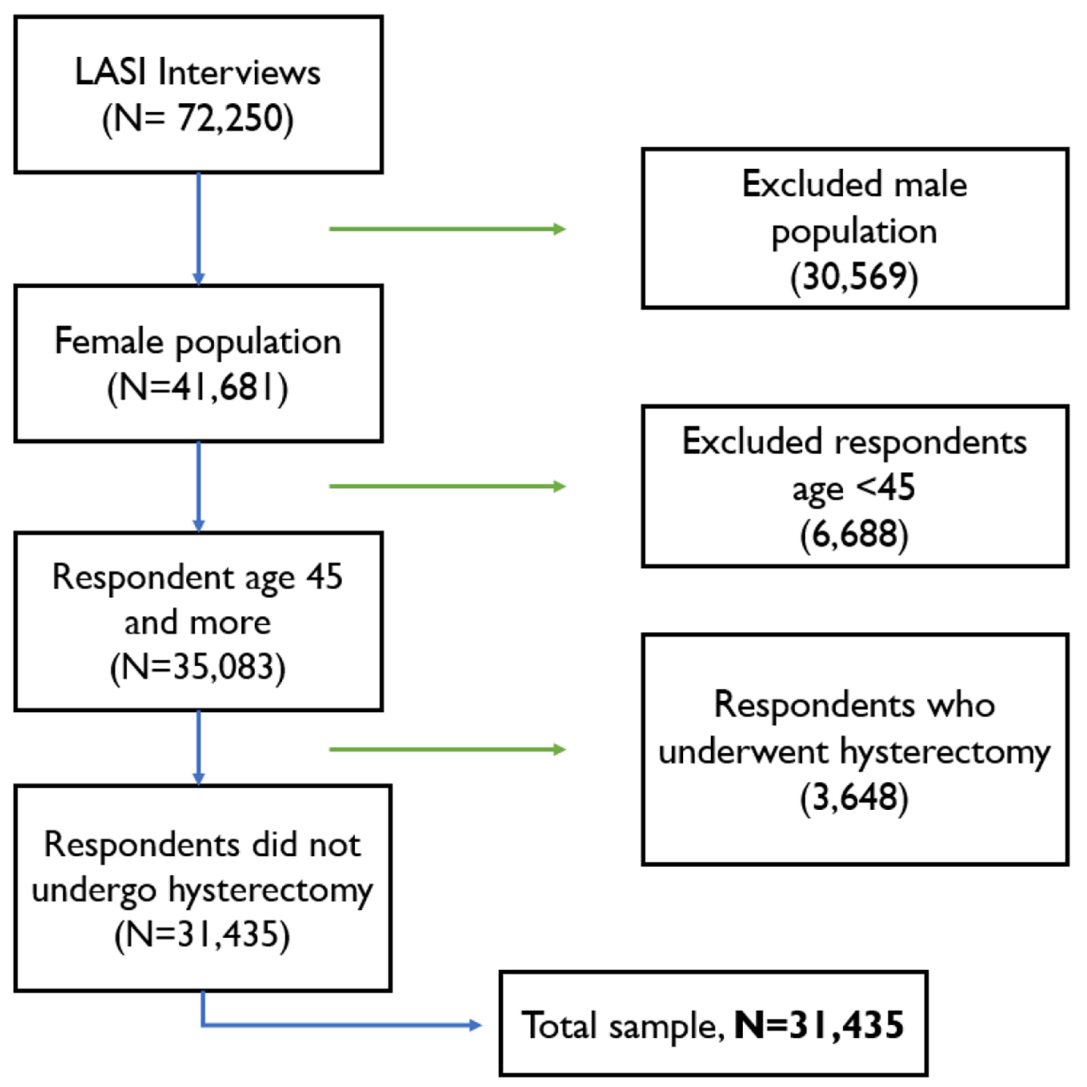
Flowchart of sample selection

The present study has used data on women aged 45 and above and did not go through hysterectomy, which is a sample of 31,435 women. Their age of menopause is computed indirectly from the available questions and measured for premature (below 30 years) and early (40 to 44 years) menopause. The flowchart of the sample selection is given in Fig. 1.

### Ethical approval

The analysis is based on publicly available secondary data. As a result, no institutional review board (IRB) permission was necessary from the researchers. The LASI was carried out in accordance with ethical standards. As part of the ethical protocols, participants were given brochures that detailed the aim of the survey, privacy protection, health assessments, and safety precautions. The consent forms followed the Human Subjects Protection Act. The data is publicly accessible via https://www.iipsindia.ac.in/content/LASI-data.

### Variable description

#### Dependent variable

Cognitive decline is a measure of ageing and a predictor of physical impairment and disability, which is likely to impact one’s capacity to work, make decisions, and interact socially [51]. Cognitive health was measured under five domains: memory, orientation, arithmetic, executive functioning, and object naming. Furthermore, a composite index was constructed as a part of the regular cognition module of the Health and Retirement Study (HRS) [52]. Overall, the cognitive score ranged from 3 to 43, where high scores indicated better cognitive function, with good reliability (Cronbach’s α = 0.93).

#### Mediators

To measure the presence of depressive symptoms, 10 Centre for Epidemiologic Studies Depression (CES-D) items were considered [53]. These items were divided into 7 negative symptoms and 3 positive symptoms, with a score ranging from 0 to 10. Furthermore, the negative symptoms were scored in ascending order, whereas the positive symptoms scoring were ranked in reverse order. Thus, high scores indicate severe depressive symptoms [52]. The Cronbach’s alpha reported is 0.85 in this analysis, indicating good reliability of the depression scale.

Insomnia is measured by four indicators, including, trouble falling asleep, waking up in the middle of the night, waking up too early, and feeling of unrest in the daytime. The categories occasionally (3–4 nights/ week) and frequently (5 or more nights per week) were considered to be insomniac. All were combined to measure insomnia and the score ranged from 0 to 4. The Cronbach’s alpha reported is 0.78 in this analysis, indicating good reliability of the insomnia indicator.

#### Independent variables

The main independent variable of the study is premature menopause and early menopause, which indicated the depletion of ovarian function. To measure the effect on late-life health, the woman’s age at menopause is checked is it is before the age of 45. Firstly, the condition is taken as the last menstrual month is more than 12 months before the survey year. The age at menopause is computed by.

*Age at Menopause = Year of menopause* - *Birth year*.

If the age at menopause is before the age of 40, then it is taken as *premature menopause*, while if the age at menopause is between 40 and 44 then it is taken as *early menopause*. Both of which are dummy variables, coded as ‘0’ and ‘1’ [54].

#### Moderator

The moderator used in the study is tobacco consumption or smoking in general. The respondent was asked about their tobacco consumption and smoking habit, and those who responded as yes were coded ‘1’ and no was coded as ‘0’.

#### Control variables

The present study adjusted socioeconomic, demographic, as well as, functional ability factors. The variables were kept as continuous or dichotomous for the convenience of the model. The age of the respondents and years of schooling were taken as continuous. The religion of the respondents was categorized as the Hindu and Other religions. The social class of the households was recoded as the schedule caste or schedule tribe, and other castes. The marital status was coded as currently married, and widowed/divorced/separated/not married.

The monthly per capita expenditure (MPCE) quintile was measured using household consumption data. Sets of 11 and 29 questions on the expenditures on food and non-food items, respectively, were used to canvas the sample households. Food expenditure was collected based on a reference period of seven days, and non-food expenditure was collected based on reference periods of 30 days and 365 days. Food and non-food expenditures have been standardized to the 30-day reference period (34). The monthly per capita consumption expenditure (MPCE) is computed and used as the summary measure of consumption. MPCE was then classified as poorest, poorer, middle, richer and richest.

In terms of health measurement, LASI has information on the prevalence of seven self-reported diagnosed chronic health conditions including, cardiovascular disease (including hypertension, heart diseases and stroke), lung disease, diabetes, bone or joint disease, psychiatric disorder and high cholesterol. The occurrence of the above diseases was coded as 0 ‘No’ and 1 ‘Yes.‘ Further, the number of cited diseases was used to calculate the ‘number of chronic diseases’ variable, which had a value ranging from 0 to 7, with a larger value suggesting more diseases.

The functional ability and mobility indicator consists of three variables, Activities of Daily Living (ADL), Instrumental Activities of Daily Living (IADL) and Mobility restriction. ADL refers to normal daily self-care activities, such as difficulty with dressing, walking across the room, bathing, eating, getting in or out of bed, or using the toilet (including getting up and down) [52]. Similarly, for the measurement of IADL, questions were asked if they were having any difficulties that were expected to last more than three months, such as preparing a hot meal, shopping for groceries, making a telephone call, taking medications, doing work around the house or garden, managing money (such as paying bills and keeping track of expenses), and getting around or finding an address in unfamiliar places [55]. Both the indices were coded dichotomous as 0 ‘No’ and 1 ‘Yes.‘ Lastly, a set of seven questions related to mobility: walking 100 yards, sitting for 2 h or more, getting up from a chair after sitting for long periods, climbing one flight of stairs without resting, stooping, kneeling or crouching, reaching or extending arms above shoulder level (either arm), pulling or pushing large objects, lifting or carrying weights over 10 pounds(such as a heavy bag of groceries), and picking up a coin from a table.

#### Statistical methods

Summary statistics was used to describe the study variables, that is, mean, standard deviations (continuous variables), frequency distribution and percentages (categorical variables). Bivariate analysis was carried out to examine the significant association between the independent variable (*X*), mediators (*M1 & M2*), moderator (*W*), control variables and the dependent variable (*Y*). Independent t-tests were used for categorical variables with two categories and one-way ANOVA F-test for more than 2 categories; whereas for continuous variables correlation tests were used. Since the control variables considered in the study could have potential confounding effects, hence they were controlled for in the moderated mediation models.

**Fig. 2:**
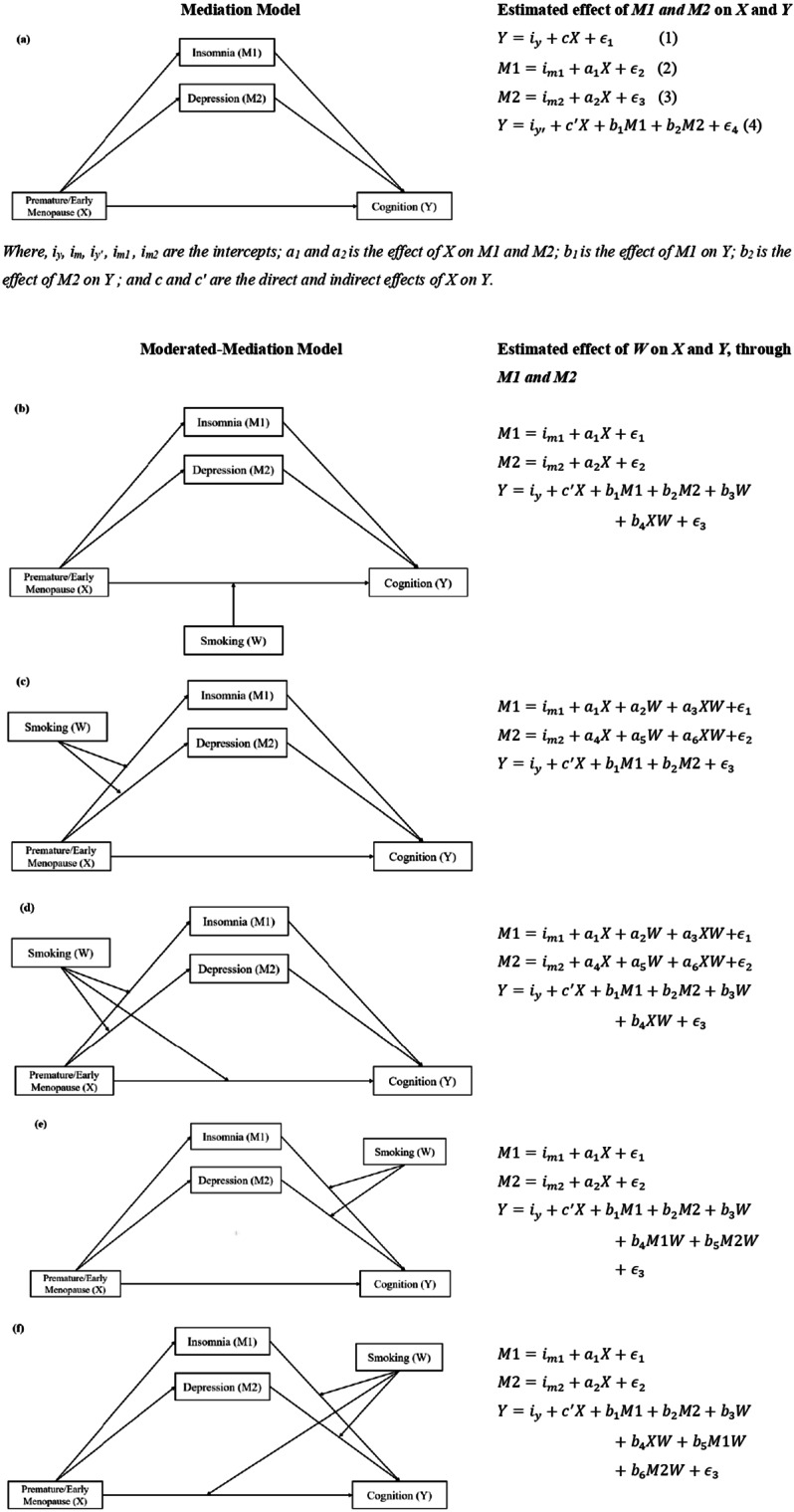
Multiple moderated mediation model, where, a_i_’s and b_i_’s are the model coefficients and the estimated effects of moderator on the indirect effect of independent variable on dependent variable through mediator, is nothing but the coefficients of the interaction terms in the models

To test the mediation and moderated mediation hypothesis, the guidelines outlined by Preacher and Hayes (2008), have been followed. Firstly, the simple mediation model (Fig. 2a) was examined for the mediation hypothesis. The model analyses the relationship between premature or early menopause (*X*) and cognition (*Y*) mediated through insomnia (*M1*) and depression (*M2*), by the following steps: (i) regressing *Y* on *X*; (ii) regressing *M1 and M2* on *X*; and (iii) regressing *Y* on *X* and *M1 & M2*. There are three requirements for mediation to exist, that includes- *a* is significant, indicating *X* is related to *M1* and *M2* (Eqs. 2 and 3); *b* is significant, indicating *M1* and *M2* is related to *Y* (Eq. 4); and if the indirect effect (a*b) *c’* is not significant, but if significant then it indicates partial mediation (Fig. 2a). This model provides direct and indirect paths among the variables controlling the possible confounders.

Then the proposed moderator variable was integrated into the model for testing the moderated mediation hypothesis. In all the other models, as shown in (Fig. 2b-f), we assessed the mediation effect of insomnia and depression moderated at categories of smoking (No, Yes), and interaction terms were computed in each model. Both the mediation and moderated mediation analysis were performed in SPSS based on 5000 bootstrapped samples (bias corrected) and sorting them to yield 95th percentile confidence interval.

## Results

Table 1 shows that, in a sample of 31,435, the mean age of the sample is 60 years and ranges from 45 to 110 years. Majority of the respondents belonged to rural areas (68.9%), belonging to Hindu religion (8.6%), and poor MPCE quintile (22%). Around 29% of the women belonged to scheduled caste or scheduled tribe (SC/ST). The average years of schooling of the sample is 2.5 years, ranging from zero to 23 years The marital status of around 61% of respondents were married and 39% unmarried/divorced/widowed/separated. The mean functional abilities indicated by ADL and IADL was 0.4 (range 0–6) and 1.28 (range 0–7) respectively. The mobility restriction score ranging from 0 to 7 has a mean score of 2.56 (SD: 2.44). The mean number of chronic diseases were 1.04 (SD: 1.12) in a range of 0 to 7.

**Table 1:** Personal data of menopause women (n = 31,435)

Covariates		N(%)	Mean(S.D)	Range
**Covariates**				
Current age			60.22 (10.98)	45–110
Residence	Rural	21,663 (68.91)		
	Urban	9772 (31.09)		
Years of schooling			2.52 (3.87)	0–23
MPCE Quintile	1 Poorest	6941 (22.08)		
	2 Poorer	6869 (21.85)		
	3 Middle	6511 (20.71)		
	4 Richer	5941 (18.9)		
	5 Richest	5173 (16.45)		
Religion	Hindu	25,655 (81.61)		
	Others	5780 (18.39)		
Caste	SC/ST	9136 (29.06)		
	Others	22,299 (70.94)		
Marital Status	Married	19,159 (60.95)		
	Unmarried/Divorced/Widow/Separated	12,276 (39.05)		
ADL			0.40(1.13)	0–6
IADL			1.28(1.98)	0–7
Mobility restriction			2.56(2.44)	0–7
No. of chronic diseases			1.04(1.12)	0–7
**Independent variable (X)**				
Premature Menopause	No	28,902 (91.94)		
	Yes	2533 (8.06)		
Early Menopause	No	27,546 (87.63)		
	Yes	3889 (12.37)		
**Mediators (M)**				
Insomnia (M1)			0.91 (1.30)	0–4
Depression(M2)			2.97 (1.77)	0–10
**Dependent variable (Y)**				
Cognition			22.42 (6.87)	3–43
**Moderator (W)**				
Tobacco Consumption	No	25,242 (80.3)		
	Yes	6193 (19.7)		

**Fig. 3:**
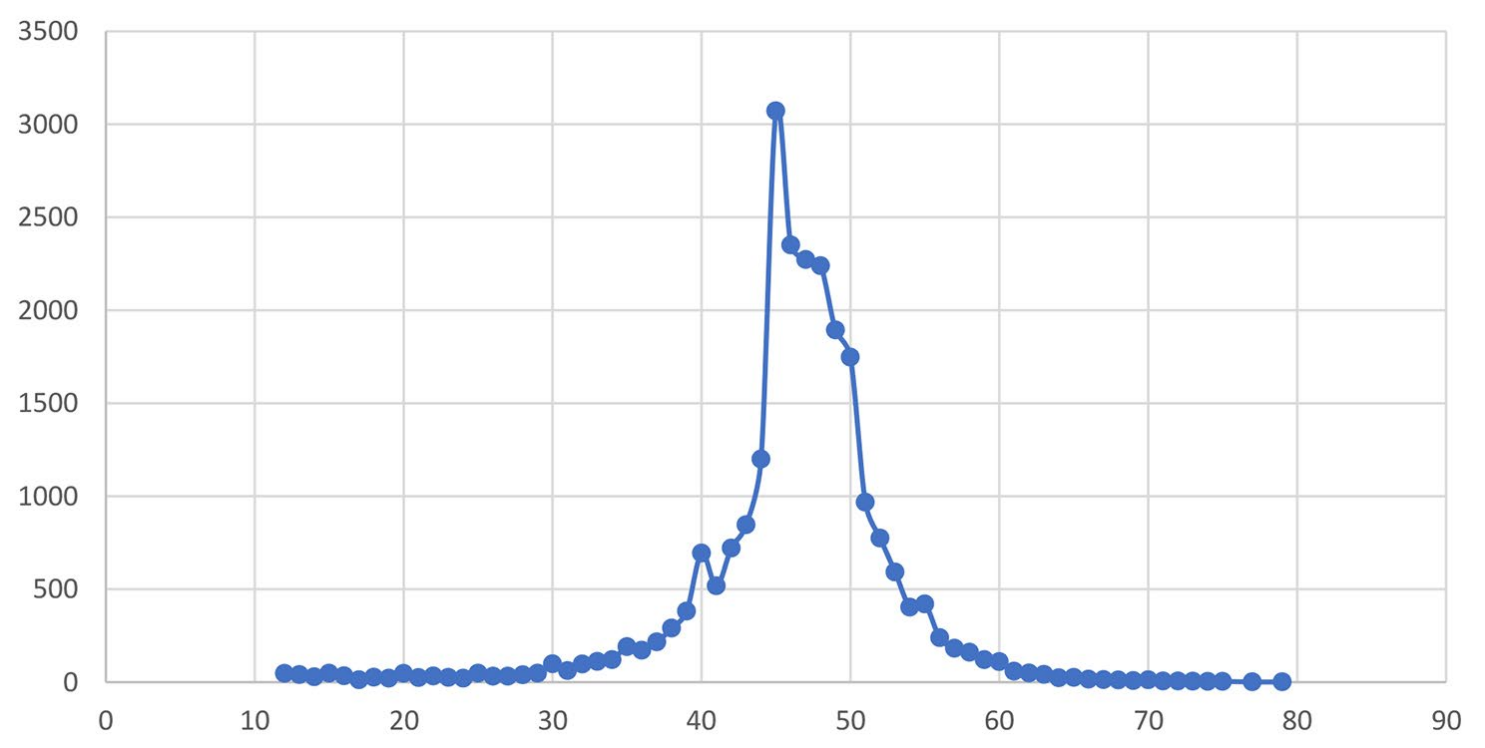
Distribution of age at menopause of the sample

The age distribution of the age at menopause of the sample from Fig. 3, shows that mainly it is concentrated at the ages between 45 and 50. Approximately 8% of the older women reported that they had prematurely menopaused (< 40 years age), while 12.4% reported having early menopause (40 to 44 years age) (Table 1).

The dependent variable, cognitive health score ranging from 3 to 43 has mean score of 22.4 (SD:6.87). The mediators depression has a mean score of 2.97 (SD: 1.77) and insomnia has a mean score of 0.9 (SD: 1.3). The moderator of the study is smoking or tobacco consumption, and 2.68% of the women.

The bivariate results are presented in Table 2, with tests of association between the study variables and dependent variable cognition. The results show that all the socioeconomic and demographic variables were significantly associated with cognition, except for religion. The age of the respondent and cognition is significantly negatively correlated (r= -0.31). The mean cognitive score was higher respondents in urban areas (26.2), not belonging to scheduled caste or scheduled tribe (24.98), and in highest MPCE quintile (25.48). Cognitive ability is also significantly positively correlated with years of schooling (r = 0.52).

**Table 2:** Bivariate tests with cognitive scores among menopause women (n = 31,435)

Covariates		Cognition (Y)		
		*Mean(S.D)*	*Test*	*p-value*
Current age			r=-0.31	< 0.001
Residence	Rural	21.38 (0.05)	t= -55.74	< 0.001
	Urban	26.18 (0.07)		
Years of schooling			r = 0.52	< 0.001
MPCE Quintile	Poorest	21.21(6.52)	F = 296.01	< 0.001
	Poorer	22.23(6.76)		
	Middle	22.95(6.98)		
	Richer	23.82(7.15)		
	Richest	25.48(7.4)		
Religion	Hindu	23.12 (0.05)	t = 0.0305	> 0.001
	Others	23.13 (0.08)		
Caste	SC/ST	21.46 (0.07)	t = 29.17	< 0.001
	Others	23.98 (0.05)		
Marital Status	Married	23.98 (0.05)	t= -26.96	< 0.001
	Unmarried/Divorced/Widow/Separated	21.58 (0.07)		
ADL			r=-0.15	< 0.001
IADL			r= -0.28	< 0.001
Mobility restriction			r= -0.16	< 0.001
No. of chronic diseases			r= -0.18	< 0.001
**Independent variable (X)**				
Premature Menopause	No	23.18 (0.04)	t = 5.39	< 0.001
	Yes	22.35 (0.15)		
Early Menopause	No	23.11 (0.05)	t= -0.90	> 0.001
	Yes	23.22 (0.11)		
**Mediators (M)**				
Insomnia (M1)			r= -0.11	< 0.001
Depression(M2)			r= -0.15	< 0.001
**Moderator (W)**				
Tobacco Consumption	No	23.25	t = 17.20	< 0.001
	Yes	19.84		

The measures of functional abilities ADL (r=-0.15) and IADL (r=-0.28), also mobility restriction (r=-0.16) showed a significant negative correlation with cognition. Depression (r=-0.15) and insomnia (r= -0.11) showed a significantly strong negative correlation with cognition. The cognitive score is significantly lower (19.84) among those who consume tobacco. Premature menopause is significantly associated with cognition and the score is lower among those who had premature menopause (Table 2).

**Table 3:** Unstandardized regression coefficients (β) with standard errors (SE)) estimating insomnia (M1), depression (M2) and cognition (Y) (adjusted for possible confounders) among menopause women (n = 31,435)

	Cognition		Insomnia		Depression		Cognition	
	β	SE			β	SE	β	SE
Constant	23.96***	0.25	0.67***	0.06	3.25***	0.08	24.74***	0.26
Premature Menopause(X)	-0.33*	0.12	0.18***	0.03	0.25***	0.04	-0.25**	0.12
Insomnia (M1)							-0.11***	0.03
Depression(M2)							-0.22***	0.02
Current age	-0.11***	0.01	-0.02*	0.01	-0.01***	0.01	-0.11***	0.01
Residence	1.53***	0.08	-0.11***	0.02	-0.17***	0.02	1.48***	0.08
Years of schooling	4.12**	0.04	-0.07***	0.01	-0.14***	0.01	4.08***	0.04
MPCE Quintile	0.26***	0.02	0.02		-0.04***	0.01	0.25***	0.03
Religion	-0.08***	0.04	-0.06***	0.01	-0.13***	0.01	-0.12**	0.04
Caste	0.22***	0.03	0.01	0.01	-0.01	0.01	0.22***	0.03
Marital Status	-0.05	0.06	0.03**	0.01	0.21***	0.02	-0.01	0.06
ADL	-0.31***	0.04	0.05***	0.01	0.12***	0.01	-0.28***	0.04
IADL	-0.23***	0.02	0.04***	0.01	0.05***	0.01	-0.21***	0.02
Mobility index	-0.08***	0.02	0.11***	0.01	0.05***	0.01	-0.07***	0.02
No. of chronic diseases	0.37***	0.03	0.09***	0.01	0.06***	0.01	0.39***	0.03
R2		0.45		0.09		0.05		0.45
F	*p < 0.001*	1746.88	*p < 0.001*	214.97	*p < 0.001*	122.18	*p < 0.001*	1516.46
Total Effect	-0.33(-0.57,-0.08)							
Direct Effect	-0.25(-0.49,-0.01)							
Indirect Effect	-0.07(-0.09,-0.05)							
Premature Menopause Insomnia Cognition	-0.02(-0.07,-0.01)							
Premature Menopause Depression Cognition	-0.05(-0.08,0.03)							

Note: Effects are significant when the upper and lower bound of the bias corrected 95% CI does not contain zero.; *** p < 0.001;** p < 0.01; *p < 0.05

**Table 4:** Coefficients of the moderated mediation models (adjusted for possible confounders) among menopause women (n = 31,435)

Model	Interaction	*Insomnia*				*Depression*				*Cognition*			
		β	SE	*R* ^*2*^	*F*	β	SE	*R* ^*2*^	*F*	β	SE	*R* ^*2*^	*F*
**B**	Premature Menopause	0.18***	0.03	0.09	233.96	0.27***	0.04	0.05	122.29	-0.38**	0.14	0.46	1425.58
	Smoking	NA				NA				-0.79***	0.08		
	Insomnia	NA								-0.12***	0.03		
	Depression	NA				NA				-0.22***	0.02		
	Premature Menopause*Smoking	NA				NA				-0.64***	0.12		
**C**	Premature Menopause	0.16***	0.05	0.09	198.25	0.25***	0.04	0.05	104.29	-0.25***	0.12	0.45	1633.18
	Smoking	0.02	0.02			-0.08**	0.03			NA			
	Insomnia	NA								-0.11***	0.03		
	Depression	NA				NA				-0.22***	0.02		
	Premature Menopause*Smoking	0.07	0.07			0.07	0.09			NA			
**D**	Premature Menopause	0.16***	0.03	0.09	198.25	0.25***	0.04	0.05	104.29	-0.38**	0.14	0.45	1425.58
	Smoking	0.02	0.02			-0.08***	0.03			-0.79***	0.08		
	Insomnia	NA								-0.12***	0.03		
	Depression	NA				NA				-0.22***	0.02		
	Premature Menopause*Smoking	0.08	0.07			0.07	0.09			-0.64***	0.12		
**E**	Premature Menopause	0.18***	0.03	0.09	233.96	0.27***	0.04	0.05	122.29	-0.26*	0.12	0.45	1336.4
	Smoking	NA				NA				-0.87***	0.16		
	Insomnia	NA				NA				-0.13***	0.03		
	Depression	NA				NA				-0.22***	0.05		
	Insomnia*Smoking	NA				NA				-0.17**	0.03		
	Depression*Smoking	NA				NA				-0.13***	0.01		
**F**	Premature Menopause	0.18***	0.03	0.09	233.96	0.27***	0.04	0.05	122.29	-0.37*	0.14	0.45	1258.09
	Smoking	NA				NA				-0.89***	0.16		
	Insomnia	NA				NA				-0.13***	0.03		
	Depression	NA				NA				-0.22***	0.02		
	Premature Menopause*Smoking	NA				NA				-0.57*	0.03		
	Insomnia*Smoking	NA				NA				-0.11**	0.06		
	Depression*Smoking	NA				NA				-0.03**	0.01		

Note: *** p < 0.001;** p < 0.01; *p < 0.05

### Depression and insomnia as mediators

**Fig. 4:**
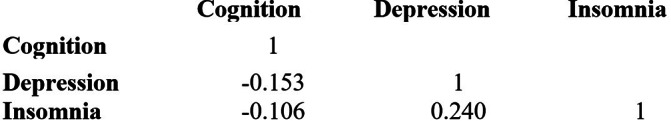
Correlation Matrix among cognition, depression and insomnia scores

The correlation matrix in Fig. 4, shows that the association between the mediators is positive, that is with increasing levels of depression, insomnia increases too, and vice versa. The relationship between premature menopause (X) and cognition (Y) has been analyzed and observed if the relationship is mediated through insomnia (M1) and depression (M2), which has been presented in Table 3. The unstandardized regression coefficients (β) with significance and standard errors (SE) have been reported. Premature ovarian failure measured by premature menopause was negatively associated with cognition (β:-0.33; SE:0.12; *p < 0.05*), whereas positively associated with insomnia (β:0.18; SE:0.03; *p < 0.001*) and depression (β:0.25; SE:0.04; *p < 0.001*). Finally, depression and premature menopause both were concurrently included in the model, where depression was negatively associated with cognitive health (β:-0.27; SE:0.02; *p < 0.001*). When the mediators are included, the negative effect of premature menopause on cognition decreases and is significant at 1% level of significance (β:-0.25; SE:0.12; *p < 0.01*). The indirect effect of premature menopause on cognition which is mediated by insomnia and depression is -0.07 and significant. This indicates partial mediation of insomnia and depression between premature menopause and cognitive health, thus resulting in 21% variability of the total effect in cognitive health.

Similarly, the model was run for early menopause as well, and the effect on cognition was not significant. Though the coefficients for insomnia and depression were significant but that does not show any mediating effect, since the main relation between independent and dependent is not significant (Table S1).

### Tobacco consumption as a moderator

**Fig. 5:**
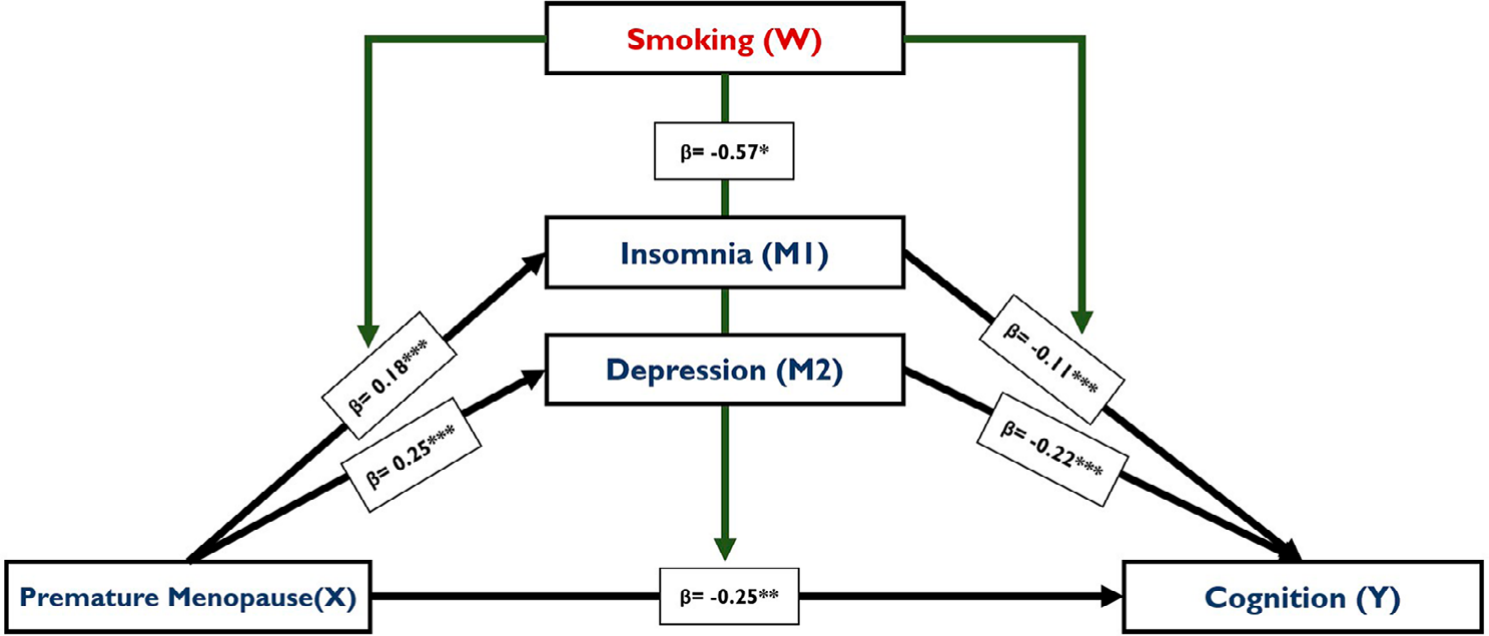
Consolidated representation of the moderated multiple mediation model; Note: *** p < 0.001; **p < 0.01; * p < 0.05

The relationship between premature menopause and cognition mediated through insomnia and depression and moderated by MPCE has been analyzed. Table 4 provides with unstandardized regression coefficients with significance and standard errors of the interaction terms in various moderated mediation models that estimated insomnia, depression and cognition. In model B and D, tobacco consumption or smoking is observed to be negatively related to cognitive function (β= -0.79; SE:0.08; *p < 0.001*), as well as in model E (β= -0.87; SE:0.16; *p < 0.001*) and F (β= -0.89; SE:0.16; *p < 0.001*). The significant effect of moderation of smoking is highest in model B and D, where all the effects in the model, including the interaction of premature menopause and smoking (β= -0.64; SE:0.12; *p < 0.001*). Model C and D shows that smoking has a significant negative association with depression (β=-0.08; SE:0.03; *p < 0.001*), although the relation of smoking and insomnia is not significant here. Interestingly, it can be observed that the interaction of moderator (smoking) with the mediators, insomnia (β=-0.17; SE:0.03; *p < 0.01*) and depression (β=-0.13; SE:0.01; *p < 0.001*), and is negatively associated with dependent variable (cognition) (Model E). In model F, the same interactions have lower negative coefficients. Overall, there is a moderating effect of smoking or tobacco consumption has a significant moderating effect in the pathways among premature menopause, depression, insomnia and cognition (Fig. 5).

Taking early menopause, the models were run similarly in this context as well, but the result was not significant in relation to early menopause and cognition (Table S2). Thus, the hypothesis for the present study is rejected in the case of early menopause.

**Fig. 6:**
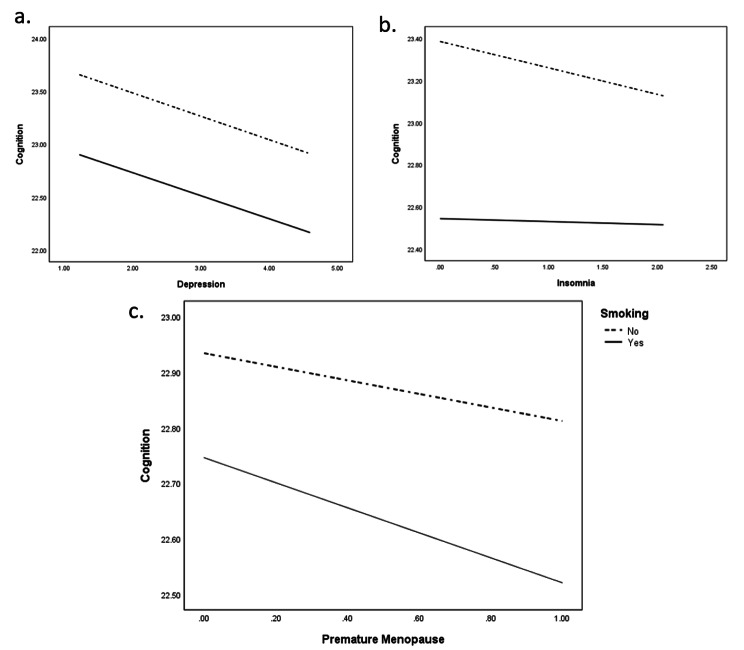
Test of moderation model indicating that the regression slopes for the two categories of smoking -No and Yes

As observed from Fig. 6a and b, showing the conditional indirect effects of premature menopause on cognition at different moderator levels (Smoking-No and Yes), there is a stronger mediation of insomnia as well as depression, among the women who do not smoke. Also, Fig. 5c shows the conditional direct effects of premature menopause on cognition at different moderator levels, where again there is a stronger mediation of both the mediators among the women who do not smoke. It can also be inferred from the plots that, with premature menopause coupled with more depression and insomnia, there is a depletion in cognitive function (Fig. 6).

## Discussion

The present study attempted to determine the effect of premature or early menopause on late-life cognitive health in India. The study hypothesized that insomnia and depression have a mediating role and that smoking or tobacco consumption has moderated the relationship between premature ovarian failure and cognitive decline. There exist limited studies using moderated mediation analysis in such public health research and this study is a major contribution to the literature. Overall, the study has shown significant results from premature menopause, while for early menopause the hypothesis was rejected. This indicates the severity of lower menopausal age and its effect on psychosocial health.

The study findings from mediation analysis indicated that insomnia and depression play a significant mediating role between premature menopause and cognitive health. Smoking was found to play a significant moderating role here, and it is worth noting that the effects were higher among smokers. The study has controlled for some of the possible confounding factors that include socio-economic and demographic factors, as well as ADL, IADL, mobility restriction and a number of chronic diseases.

The direct effect of a premature menopause on cognitive health, without any mediators or moderators, and controlling for the possible confounders, have shown that there is a negative impact. There have been no studies that focussed on how premature menopause can affect the cognitive function of women in the Indian context. Overall, too there exists scanty literature exploring this association. Among these studies, the results showed that earlier age at menopause leads to an earlier decline in cognitive function, which remains consistent with the current study results [13, 56].

### The mediating role of insomnia and depression

A woman experiencing menopause have decreased production of estrogen which leads to symptoms of sleep problems or insomnia along with other prominent one like vaginal dryness, hot flushes, joint pain etc. There are also psychological symptoms like irritations, anxieties, extreme mood swings, depression and emotional turbulences [57]. Now when a woman is experiencing menopause at an early age, before 40s then it is due to premature ovarian insufficiency, and the aforementioned symptoms also occur to them.

A study by Ates et al. (2022), which is based on 62 women with premature ovarian insufficiency and 62 controls, in Turkey, found poor sleep quality and insomnia among the premature menopausal women than the controls [58]. The severity of depression and anxiety was also high among premature menopausal women. The present study findings also suggest an effect of a premature menopause on insomnia and depression, and the relationship is positively associated. There also exists a strong mediation effect of insomnia and depression between premature menopause and cognitive decline. Women with premature ovarian failure also have low sex steroid levels and higher gonadotropic levels [59, 60]. They have more anxiety, depression, sensitivity and psychological distress [61]. All of this cumulatively affects cognitive health, and there is an increased risk of premature menopausal women of developing cognitive impairment or dementia [1, 62].

### The moderating role of smoking

The present research constructed a moderated multiple mediation model for testing whether insomnia and depression mediated the relation between premature menopause and cognitive health, and whether this indirect relationship is moderated by smoking habit. The results indicated that smoking moderated this indirect link and the study hypothesis is accepted.

Cigarette smoking as a lifestyle factor has been consistently linked with earlier age at spontaneous menopause in previous studies. Smoking has an anti-oestrogenic impact due to the increased production of adrenal androgens, which leads to resistance of the oestrogen functions [63–66]. This overall jeopardizes the normal female hormonal balance, which affects the overall system. When smoking effect interacts with being prematurely menopaused, hormonal imbalances lead to sleep disturbances, cause depressive symptoms and ultimately affect cognitive function [67, 68].

### Strengths and Limitations of the study

The study has several strengths owing to its national representativeness and methodological robustness. This study controlled for most of the factors that could confound the results. The use of the statistical tool ‘PROCESS’ which allows the analysis of mediation, moderation and moderated mediation models, is very less in the Indian context and especially in public health research, and this study adds on to it. However, the cross-sectional nature of the data limits the establishment of more causal relationships. Mental health issues and sleep problems can occur anytime due to other reasons as well. A longitudinal study in future in this context can yield better results.

## Conclusion

The study is the first attempt in carrying out multiple mediations and moderated analysis to show the effect of premature ovarian failure on cognitive health, mediated through insomnia and depression and moderated by smoking habit. The findings emphasize the fact that smoking is associated with premature menopause, depression and insomnia. Women who experienced premature menopause has lower cognitive scores, depressive symptoms and insomnia symptoms, which were higher among those who consumed tobacco.

### Practice recommendations

There needs to be more awareness and research on the topic of early onset of menopause. The study, strongly recommends the dissemination of information on the negative effects of tobacco consumption and making more informed choices to maintain a healthy life. More research into various methods and therapy is needed to determine the relationship between the age of early menopause and psychosocial well-being. Public awareness and education are crucial tools for maintaining healthy lives. However, greater study on the repercussions of premature or early menopause is strongly advised, given there are relatively few studies available in the Indian setting.

### Electronic supplementary material

Below is the link to the electronic supplementary material.

Supplementary Material 1

## Data Availability

The analysis is based on secondary data available in public domain for research; thus, no approval was required from any institutional review board (IRB). The data is freely available upon request from https://iipsindia.ac.in/sites/default/files/LASI_DataRequestForm_0.pdf. **Point of contact**: Sampurna Kundu, Email: sampurna34@gmail.com.
