# Supplementary material for: Linkage of premature and early menopause with psychosocial well-being: a moderated multiple mediation approach
Source: BMC Psychol. 2023 Aug 9;11:228. doi: 10.1186/s40359-023-01267-3 (PMC10413596; doi:10.1186/s40359-023-01267-3)
Supplement: Supplementary file 1 — Supplementary Material 1 [file 40359_2023_1267_MOESM1_ESM.docx]

**Table S1:** Unstandardized regression coefficients (β) with standard errors (SE)) estimating insomnia (M1), depression (M2) and cognition (Y) (adjusted for possible confounders)

|  | **Cognition** | | **Insomnia** | | **Depression** | | **Cognition** | |
| --- | --- | --- | --- | --- | --- | --- | --- | --- |
|  | **β** | **SE** |  |  | **β** | **SE** | **β** | **SE** |
| Constant | 23.89*** | 0.25 | 0.68*** | 0.06 | 3.32*** | 0.08 | 24.68*** | 0.27 |
| Early Menopause(X) | 0.13 | 0.09 | 0.04** | 0.02 | 0.07** | 0.03 | 0.15 | 0.1 |
| Depression(M1) |  |  |  |  |  |  | -0.22*** | 0.02 |
| Insomnia (M2) |  |  |  |  |  |  | -0.12*** | 0.03 |
| Current age | -0.11*** | 0.01 | -0.01 | 0.01 | -0.02 | 0.01 | -0.11*** | 0.04 |
| Residence | 1.53*** | 0.08 | 0.11*** | 0.02 | -0.15*** | 0.02 | 1.48*** | 0.07 |
| Years of schooling | 4.12*** | 0.04 | -0.06*** | 0.01 | -0.14*** | 0.01 | 4.08*** | 0.04 |
| MPCE Quintile | 0.26*** | 0.03 | 0.01* | 0.01 | -0..04 | 0.01 | 0.25*** | 0.03 |
| Religion | -0.08*** | 0.04 | -0.07*** | 0.01 | -0.13*** | 0.01 | -0.12** | 0.04 |
| Caste | 0.22*** | 0.03 | 0.01 | 0.08 | -0.01 | 0.01 | 0.22*** | 0.03 |
| Marital Status | -0.05 | 0.06 | 0.03** | 0.01 | 0.21*** | 0.02 | -0.01 | 0.06 |
| ADL | -0.31*** | 0.04 | 0.05*** | 0.01 | 0.12*** | 0.01 | -0.28*** | 0.04 |
| IADL | -0.23*** | 0.02 | 0.04*** | 0.01 | 0.05*** | 0.01 | -0.21*** | 0.02 |
| Mobility index | -0.09*** | 0.02 | 0.10*** | 0.01 | 0.05*** | 0.01 | -0.07*** | 0.02 |
| No. of chronic diseases | 0.37*** | 0.03 | 0.09*** | 0.01 | 0.06*** | 0.01 | 0.39*** | 0.03 |
| R2 |  | 0.45 |  | 0.09 |  | 0.05 |  | 0.45 |
| F | p<0.001 | 1904.75 | p<0.001 | 233.96 | p<0.001 | 118.38 | p<0.001 | 1632.9 |
|  |  |  |  |  |  |  |  |  |
| Total Effect | 0.13(-0.07,0.32) | |  |  |  |  |  |  |
| Direct Effect | 0.15(-0.05,0.34) | |  |  |  |  |  |  |
| Indirect Effect | -0.03(-0.04,-0.01) | |  |  |  |  |  |  |
| Premature Menopause --> Insomnia --> Cognition | -0.01(-0.02,0.00) | |  |  |  |  |  |  |
| Premature Menopause --> Depession --> Cognition | -0.02(-0.03,-0.01) | |  |  |  |  |  |  |
| *Note: Effects are significant when the upper and lower bound of the bias corrected 95% CI does not contain zero.* | | | |  |  |  |  |  |

**Table S2:** Coefficients of the moderated mediation models (adjusted for possible confounders)

| **Model** | **Interaction** | ***Insomnia*** |  |  |  | ***Depression*** |  |  |  | ***Cognition*** |  |  |  |
| --- | --- | --- | --- | --- | --- | --- | --- | --- | --- | --- | --- | --- | --- |
|  |  | **β** | **SE** | ***R^2^*** | ***F*** | **β** | **SE** | ***R^2^*** | ***F*** | **β** | **SE** | ***R^2^*** | ***F*** |
|  |  |  |  |  |  |  |  |  |  |  |  |  |  |
| **B** | Early Menopause | 0.04* | 0.02 | 0.09 | 230.31 | 0.04** | 0.03 | 0.05 | 118.38 | 0.14 | 0.11 | 0.46 | 1424.82 |
|  | Smoking | NA |  |  |  | NA |  |  |  | -0.74*** | 0.08 |  |  |
|  | Insomnia | NA |  |  |  | NA |  |  |  | -0.11*** | 0.03 |  |  |
|  | Depression | NA |  |  |  | NA |  |  |  | -0.22*** | 0.02 |  |  |
|  | Early Menopause*Smoking | NA |  |  |  | NA |  |  |  | 0.01 | 0.25 |  |  |
|  |  |  |  |  |  |  |  |  |  |  |  |  |  |
| **C** | Early Menopause | 0.04 | 0.03 | 0.09 | 195.08 | 0.08** | 0.03 | 0.05 | 100.99 | 0.15 | 0.09 | 0.45 | 1632.9 |
|  | Smoking | 0.03 | 0.02 |  |  | -0.07** | 0.03 |  |  | NA |  |  |  |
|  | Insomnia | NA |  |  |  |  |  |  |  | -0.11*** | 0.03 |  |  |
|  | Depression | NA |  |  |  | NA |  |  |  | -0.22*** | 0.02 |  |  |
|  | Early Menopause*Smoking | 0.03 | 0.06 |  |  | -0.06 | 0.08 |  |  | NA |  |  |  |
|  |  |  |  |  |  |  |  |  |  |  |  |  |  |
| **D** | Early Menopause | 0.04 | 0.03 | 0.09 | 195.08 | 0.08** | 0.03 | 0.05 | 100.99 | 0.14 | 0.11 | 0.45 | 1424.82 |
|  | Smoking | 0.03 | 0.02 |  |  | -0.07** | 0.03 |  |  | -0.74*** | 0.09 |  |  |
|  | Insomnia | NA |  |  |  |  |  |  |  | -0.11*** | 0.03 |  |  |
|  | Depression | NA |  |  |  | NA |  |  |  | -0.22*** | 0.02 |  |  |
|  | Early Menopause*Smoking | 0.03 | 0.06 |  |  | -0.06 | 0.08 |  |  | 0.01 | 0.25 |  |  |
|  |  |  |  |  |  |  |  |  |  |  |  |  |  |
| **E** | Early Menopause | 0.04* | 0.02 | 0.09 | 230.31 | 0.07** | 0.03 | 0.05 | 118.38 | 0.14 | 0.09 | 0.45 | 1336.11 |
|  | Smoking | NA |  |  |  | NA |  |  |  | -0.87*** | 0.16 |  |  |
|  | Insomnia | NA |  |  |  | NA |  |  |  | -0.13*** | 0.03 |  |  |
|  | Depression | NA |  |  |  | NA |  |  |  | -0.22*** | 0.02 |  |  |
|  | Insomnia*Smoking | NA |  |  |  | NA |  |  |  | 0.12* | 0.06 |  |  |
|  | Depression*Smoking | NA |  |  |  | NA |  |  |  | 0.01 | 0.05 |  |  |
|  |  |  |  |  |  |  |  |  |  |  |  |  |  |
| **F** | Early Menopause | 0.07* | 0.03 | 0.09 | 88.09 | 0.10* | 0.05 | 0.04 | 38.65 | 0.1 | 0.17 | 0.31 | 265.14 |
|  | Smoking | NA |  |  |  | NA |  |  |  | -1.02** | 0.28 |  |  |
|  | Insomnia | NA |  |  |  | NA |  |  |  | -0.18** | 0.05 |  |  |
|  | Depression | NA |  |  |  | NA |  |  |  | -0.19*** | 0.03 |  |  |
|  | Early Menopause*Smoking | NA |  |  |  | NA |  |  |  | 0.31 | 0.44 |  |  |
|  | Insomnia*Smoking | NA |  |  |  | NA |  |  |  | 0.07 | 0.12 |  |  |
|  | Depression*Smoking | NA |  |  |  | NA |  |  |  | 0.12 | 0.09 |  |  |
| ***Note:*** *Effects are significant when the upper and lower bound of the bias corrected 95% CI does not contain zero.* | | | | |  |  |  |  |  |  |  |  |  |
| **** p<0.001, ** p<0.01, * p<0.05* | |  |  |  |  |  |  |  |  |  |  |  |  |
